# Sustaining Control of Schistosomiasis Mansoni in Western Côte d’Ivoire: Results from a SCORE Study, One Year after Initial Praziquantel Administration

**DOI:** 10.1371/journal.pntd.0004329

**Published:** 2016-01-20

**Authors:** Rufin K. Assaré, Yves-Nathan T. Tian-Bi, Patrick K. Yao, Nicaise A. N’Guessan, Mamadou Ouattara, Ahoua Yapi, Jean T. Coulibaly, Aboulaye Meïté, Eveline Hürlimann, Stefanie Knopp, Jürg Utzinger, Eliézer K. N’Goran

**Affiliations:** 1 Swiss Tropical and Public Health Institute, Basel, Switzerland; 2 University of Basel, Basel, Switzerland; 3 Unité de Formation et de Recherche Biosciences, Université Félix Houphouët-Boigny, Abidjan, Côte d’Ivoire; 4 Centre Suisse de Recherches Scientifiques en Côte d’Ivoire, Abidjan, Côte d’Ivoire; 5 Programme National de Lutte contre la Schistosomiase, les Géohelminthiases et la Filariose Lymphatique, Abidjan, Côte d’Ivoire; 6 Wolfson Wellcome Biomedical Laboratories, Department of Life Sciences, Natural History Museum, London, United Kingdom; George Washington University, UNITED STATES

## Abstract

**Background:**

The Schistosomiasis Consortium for Operational Research and Evaluation (SCORE) has launched several large-scale trials to determine the best strategies for gaining and sustaining control of schistosomiasis and transitioning toward elimination. In Côte d’Ivoire, a 5-year cluster-randomized trial is being implemented in 75 schools to sustain the control of schistosomiasis mansoni. We report *Schistosoma mansoni* infection levels in children one year after the initial school-based treatment (SBT) with praziquantel and compare with baseline results to determine the effect of the intervention.

**Methodology:**

The baseline cross-sectional survey was conducted in late 2011/early 2012 and the first follow-up in May 2013. Three consecutive stool samples were collected from 9- to 12-year-old children in 75 schools at baseline and 50 schools at follow-up. Stool samples were subjected to duplicate Kato-Katz thick smears. Directly observed treatment (DOT) coverage of the SBT was assessed and the prevalence and intensity of *S*. *mansoni* infection compared between baseline and follow-up.

**Principal Findings:**

The *S*. *mansoni* prevalence in the 75 schools surveyed at baseline was 22.1% (95% confidence interval (CI): 19.5–24.4%). The DOT coverage was 84.2%. In the 50 schools surveyed at baseline and one year after treatment, the overall prevalence of *S*. *mansoni* infection decreased significantly from 19.7% (95% CI: 18.5–20.8%) to 12.8% (95% CI: 11.9–13.8%), while the arithmetic mean *S*. *mansoni* eggs per gram of stool (EPG) among infected children slightly increased from 92.2 EPG (95% CI: 79.2–105.3 EPG) to 109.3 EPG (95% CI: 82.7–135.9 EPG). In two of the 50 schools, the prevalence increased significantly, despite a DOT coverage of >75%.

**Conclusions/Significance:**

One year after the initial SBT, the *S*. *mansoni* prevalence had decreased. Despite this positive trend, an increase was observed in some schools. Moreover, the infection intensity among *S*. *mansoni*-infected children was slightly higher at the 1-year follow-up compared to the baseline situation. Our results emphasize the heterogeneity of transmission dynamics and provide a benchmark for the future yearly follow-up surveys of this multi-year SCORE intervention study.

## Author Summary

Schistosomiasis is a parasitic worm disease that is widespread in sub-Saharan Africa. To better understand how to gain and sustain the control of schistosomiasis and how to eliminate this disease in different epidemiologic settings, the Schistosomiasis Consortium for Operational Research and Evaluation (SCORE) has launched several multi-year studies that are being implemented in East and West Africa. This article highlights how the *Schistosoma mansoni* infection levels changed one year after an initial treatment with the anti-worm drug praziquantel given to children aged 5–15 years in western Côte d’Ivoire. Infection and treatment data at school level were available from more than 4,600 children in 50 schools. One year after the treatment that had been received by more than 80% of the children, the overall *S*. *mansoni* prevalence decreased from 19.7% to 12.8%, while the intensity of infection among *S*. *mansoni*-positive children slightly increased. In several schools, the *S*. *mansoni* intensity and, particularly the prevalence, increased unexpectedly. Our findings show that the dynamics of schistosomiasis transmission varies from one village to another. It will be interesting to monitor changes over longer time periods as this SCORE study unfolds.

## Introduction

Schistosomiasis is a neglected tropical disease that exerts a considerable public health problem in 78 tropical and subtropical countries [1]. In 2013, it was estimated that schistosomiasis affected more than 250 million people worldwide with 90% of the reported cases concentrated in sub-Saharan Africa [2]. Since the mid-1980s, the World Health Organization (WHO) emphasizes morbidity control using the drug praziquantel as the main pillar of the global strategy to fight schistosomiasis [3]. Praziquantel is the drug of choice because it is efficacious against the adult stages of all *Schistosoma* species parasitizing humans, is inexpensive (the average cost to treat a school-aged child was US$ 0.2 per treatment in 2013), and has a good safety profile [4–8]. For morbidity control, praziquantel is being administered to at-risk populations without prior diagnosis, a strategy commonly known as ‘preventive chemotherapy’ [9].

The recommended frequency of drug administration is based on the level of endemicity in a given study area. According to WHO, in areas with high schistosomiasis endemicity (prevalence ≥50%), all school-aged children and adult people at risk of infection should be treated annually [10]. In areas with moderate endemicity (prevalence 10–50%), all school-aged children should be treated once every two years. In low endemic areas (prevalence <10%), school-aged children should be treated twice during their time in school; first at school entry and then again in their last year of schooling [11,12]. However, these prevalence thresholds are arbitrary. Hence, the Schistosomiasis Consortium for Operational Research and Evaluation (SCORE) launched a series of studies to strengthen the evidence-base how best to gain and sustain the control of schistosomiasis, including cost considerations [13]. Two 5-year cluster-randomized trials are being implemented in Côte d’Ivoire and Kenya [14,15]. These trials are school-based with three treatment arms (25 schools per arm) and aim to assess whether annual school-based treatment (SBT) with praziquantel for four years (arm A), annual SBT in years 1 and 2, followed by “drug holidays” in years 3 and 4 (arm B), or SBT in years 1 and 3, spaced by“drug holidays” in years 2 and 4 (arm C) will substantially reduce the prevalence and intensity of *Schistosoma* infection and keep infection at low levels.

Here, we present the effect of the first SBT with praziquantel on *Schistosoma mansoni* infection among school-aged children in western Côte d’Ivoire, as revealed by a detailed follow-up survey conducted in May 2013, compared to baseline data collected from December 2011 to February 2012. Specifically, we determined changes in the prevalence and intensity of *S*. *mansoni* infections among children in the 50 schools that belong to treatment arms A and B, and discuss consequences for the ongoing cluster-randomized trial and, more generally, for schistosomiasis control interventions in Côte d’Ivoire and elsewhere.

## Methods

### Ethics Statement

The study protocol was approved by the institutional research commissions of the Swiss Tropical and Public Health Institute (Basel, Switzerland) and the ‘Centre Suisse de Recherches Scientifiques en Côte d’Ivoire’ (CSRS; Abidjan, Côte d’Ivoire). Ethical approval was obtained from the ethics committees in Basel (reference no. EKBB 279/10) and the Ministry of Public Health in Côte d’Ivoire (reference no. 1994 MSHP/CNER).

At the onset of the study, regional directors of the education and health sectors, education inspectors, village authorities, local community members, and teachers were sensitized in detail about the objectives of the research project. Parents and guardians of study participants provided written informed consent for children to participate. After the baseline parasitologic survey, in the frame of the first SBT conducted in June 2012, school-aged children living in the catchment area of participating schools were offered treatment with praziquantel at a single oral dose of 40 mg/kg of body weight [16].

### Study Area and Population

The baseline survey was carried out from December 2011 to February 2012, the SBT in June 2012, and the first follow-up survey was conducted in May 2013 in eligible schools located in four regions of western Côte d’Ivoire: Cavally, Guemon, Haut-Sassandra, and Tonkpi. Details of the study area and population surveyed have been described elsewhere [15,17]. The Cavally and Sassandra rivers and their tributaries represent the major hydrographic features of the study area [18,19]. Buyo, a hydroelectric dam built across the Sassandra River in 1981, formed a lake with an estimated surface area of 600 km² [20]. In western Côte d’Ivoire, the sources of water are traditional wells, rain water, rivers, water supply dams, ponds, creeks, fountains, natural spring water, and tap water [21]. The main reasons for human water contact that might lead to schistosomiasis transmission are washing dishes, washing children, fetching water, fishing, swimming, farming, and playing [22]. Despite the existence of latrines in numerous households, open defecation is commonly practiced [22–24].

### Sample Size

The aim of the SCORE sustaining schistosomiasis control study implemented in western Côte d’Ivoire is to determine the best strategy of preventive chemotherapy with praziquantel to sustain schistosomiasis mansoni control in moderate endemicity settings [15,17]. For this purpose, the *S*. *mansoni* prevalence in *n* schools in three treatments arms is compared over a study period of four years. The prevalence of *S*. *mansoni* is determined by testing *m* children in those schools where there is subsequent treatment. The effect of the different treatment intervals on the *S*. *mansoni* prevalence will be estimated using the following logistic regression model: *log (p*_*ijt*_ */ (1 − p*_*ijt*_*)) = μ +α*_*i*_ *+ β*_*t*_ *+ ɣ*_*ik*_, where *p*_*ijt*_ denotes the prevalence of *S*. *mansoni* in school *j* receiving treatment *i* in year *t*, *μ* is an intercept term, *α*_*i*_ is the effect of treatment *i*, *β*_*t*_ is the effect of time *t*, and γ_*ik*_ is the time by treatment interaction. Generalized estimating equations have been used to fit these longitudinal data [25]. To take into account variation in the *S*. *mansoni* prevalence among schools, an overdispersion parameter *φ* was introduced into the model. When *φ* = 1, all schools under the same treatment have identical prevalences, whereas *φ* increases with increasing variation of prevalence levels between villages.

The calculations revealed that studying 20 schools per arm and evaluating 100 individuals per school would result in minimum effect sizes of 5–12% with or without overdispersion. In order to increase the chance of detecting differences between the intervention arms, the number of intervention units was increased to 25 per arm. Consequently, a total of 75 schools with a *S*. *mansoni* prevalence of 10–24% according to results from an eligibility survey were randomized to one of the three treatment arms [15]. Treatment arm A receives SBT with praziquantel once every year for four years, arm B receives SBT in years 1 and 2, followed by “drug holidays” in years 3 and 4, and arm C receives SBT in years 1 and 3, alternated by “drug holidays” in years 2 and 4 [15]. Before administration of the first round of treatment, a detailed baseline survey was conducted.

### Study Procedures

Following the SCORE harmonization protocol, all 75 schools were included in the baseline parasitologic survey implemented in Côte d’Ivoire from December 2011 to February 2012. Children were treated with praziquantel in June 2012. Only the 50 schools belonging to treatment arms A and B were subjected to the first follow-up survey carried out in May 2013, while the 25 schools belonging to treatment arm C were not subjected to a follow-up survey, as they were on “drug holidays” in year 2.

Baseline and follow-up surveys pursued cross-sectional designs. Study procedures have been detailed elsewhere [15,17]. In brief, in each of the selected schools, approximately 100 children were invited to participate in the study. Inclusion criteria were as follows: (i) age of children ranging between 9 and 12 years; (ii) presence of an informed consent sheet signed by parents/guardians; and (iii) children themselves assented orally. Over three consecutive days, children were invited to submit a portion of their own morning stool in a 125-ml plastic container. Every day, filled stool containers were collected by trained field enumerators and sent to the hospital laboratories in the towns of Biankouma, Danané, Douékoué, Guiglo, Kouibly, and Man for processing.

Stool specimens were subjected to the Kato-Katz method [26]. In brief, duplicate Kato-Katz thick smears were prepared from a single stool sample, using 41.7 mg plastic templates. The thick smears were allowed to clear for at least 60 min and examined by experienced laboratory technicians under a light microscope at low magnification. Eggs from *S*. *mansoni*, and additionally from soil-transmitted helminth species, were counted and recorded for each species separately. For quality control, 10% of the slides were randomly selected and re-read by a senior microscopist. In case of discrepancies, the results were discussed with the concerned microscopists and the slides re-read until agreement was reached [27].

### Praziquantel Administration

In June 2012, children aged 5–15 years enrolled in the 75 study schools and non-enrolled school-aged children living in the school catchment areas were offered free-of-charge treatment with praziquantel (40 mg/kg) using a dose pole according to WHO guidelines [16]. Praziquantel was administered by trained teachers to children, following a directly observed treatment (DOT) approach. Children remained under medical observation and adverse events were recorded within 4 hours post-treatment. Treatment was led by the ‘Programme National de Lutte contre la Schistosomiase, les Géohelminthiases et la Filariose Lymphatique’ (PNL-SGF), and supported by staff from the ‘Programme National de Santé Scolaire et Universitaire’ (PNSSU), the CSRS, and the ‘Université Félix Houphouët-Boigny’. Praziquantel tablets were supplied by the Schistosomiasis Control Initiative (SCI; London, United Kingdom).

The overall number of school-aged children residing in each village was obtained by adding up the number of school-aged but non-school attending children as recorded by the community health workers and the number of children registered in school, as detailed by school teachers. Trained teachers administered praziquantel to children (those attending school, and the non-enrolled children) and recorded the number of treated children.

### Statistical Analysis

Baseline survey data were entered into Microsoft Excel (2010 Microsoft Corporation), while data from the first follow-up survey were directly entered into smartphones and then uploaded to a database maintained on a central server (Open Data Kit) in Atlanta, United States of America. Statistical analyses were performed with STATA version IC13.1 (Stata Corporation; College Station, United States of America). The final analysis included children aged 9–12 years who had at least four Kato-Katz thick smear readings at the parasitologic surveys done both at baseline and follow-up. To obtain individuals’ eggs per gram of feces (EPG), we divided the total *S*. *mansoni* egg counts from the multiple Kato-Katz slides per child by the total number of Kato-Katz thick smears and multiplied by a factor of 24. *S*. *mansoni*-positive individuals were stratified into three infection intensity categories: (i) light (1–99 EPG), (ii) moderate (100–399 EPG), and (iii) heavy (≥400 EPG) [16]. Moreover, we calculated *S*. *mansoni* prevalence and arithmetic mean (AM) EPG for positive individuals per school and treatment arm. With regard to soil-transmitted helminth infections that were also identified with the Kato-Katz technique, a child was considered positive if at least one egg of *Ascaris lumbricoides*, hookworm, or *Trichuris trichiura* was detected in one of the slides.

We employed a χ² test to assess a potential association between *S*. *mansoni* prevalence and age or sex. Reduction in the prevalence and intensity of *S*. *mansoni* infection per school was calculated using the following formulae [28]: prevalence reduction = [(prevalence at baseline—prevalence at first follow-up) / (prevalence at baseline)] X 100. Reduction in the intensity of infection = [(AM EPG at baseline—AM EPG at first follow-up) / (AM EPG at baseline)] X 100.

The treatment coverage rate was assessed by using the following formula: coverage of the mass drug administration (MDA) = [(number of school-aged children with DOT recorded by teachers) / (overall number of school-aged children registered in school and recorded by health workers)] X 100.

Geographic coordinates of each school were recorded using a hand-held global positioning system (GPS) receiver (Garmin Etrex 30; Olathe, United States of America). Arc Map 10.2.1 (Environmental Systems Research Institute Inc.; Redlands, United States of America) was used to generate maps of the changes of *S*. *mansoni* prevalence and intensity of infection (AM EPG) from baseline to follow-up.

## Results

### Operational Results from the Baseline Survey

**Fig 1:**
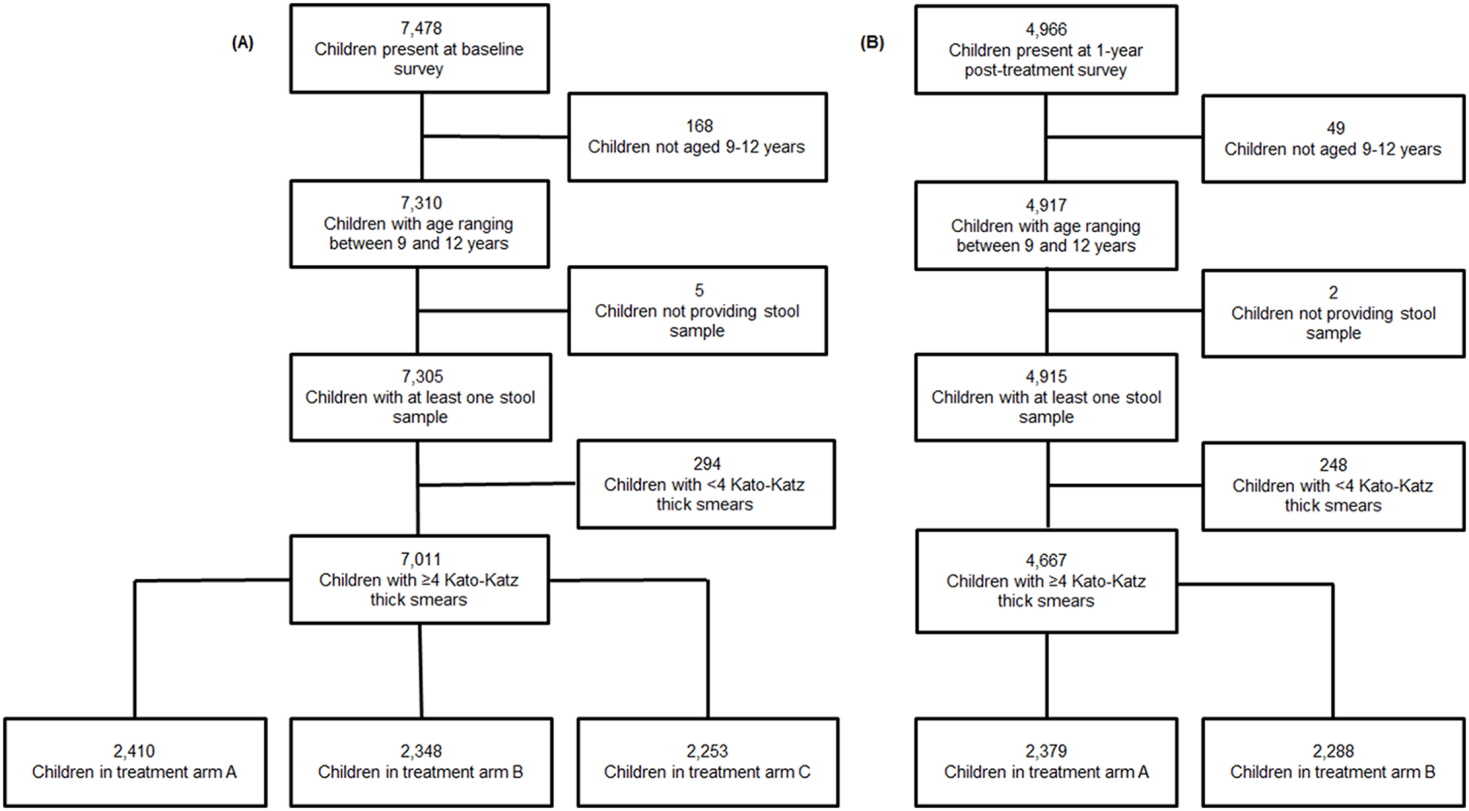
Study participation of schoolchildren at the baseline survey and one-year follow-up survey. The flowcharts show the study participation of 9- to 12-year-old schoolchildren at the baseline survey (A), which was conducted from December 2011 to February 2012, and the first follow-up survey (B), which was carried out one-year post-treatment in May 2013, in western Côte d’Ivoire.

The baseline survey was conducted in the 75 schools meeting eligibility criteria from December 2011 to February 2012, and 7,478 children were invited to participate (Fig 1). Among them, 168 pupils were excluded from further analyses, because their age was outside the 9–12 years range. Additionally, 299 children were excluded because they did not provide sufficient stool to prepare at least quadruplicate Kato-Katz thick smears. The final study population for analysis of the baseline survey consisted of 7,011 children. There were more boys (n = 4,173) than girls (n = 2,838). The mean age was 10.5 years. The number of children in treatment arms A, B, and C was 2,410 (34.4%), 2,348 (33.5%), and 2,253 (32.1%), respectively.

### Operational Results from Follow-Up Survey

In May 2013, 4,966 children from the 50 schools belonging to intervention arms A and B were invited to participate in the first follow-up survey. According to the SCORE harmonization protocol, children attending schools belonging to study arm C were not surveyed. Among the pupils attending schools included in arms A and B, who were invited to participate, 49 children had an age outside the 9–12 years range, and 250 children did not provide enough stool for at least quadruplicate Kato-Katz thick smears. Hence, results of 4,667 children were included for further statistical analyses. There were more boys (n = 2,640) than girls (n = 2,027). The children’s mean age was 10.3 years. There were 2,379 children in treatment arm A and 2,288 in treatment arm B.

### *S*. *mansoni* Infection at Baseline

At baseline, before the implementation of the first SBT with praziquantel, the examination of at least four Kato-Katz thick smears per child revealed an overall *S*. *mansoni* prevalence of 22.1% among the 75 schools surveyed. The prevalence at the unit of the school ranged from 1.0% to 54.0%. *S*. *mansoni* infection was significantly associated with age (χ² = 25.2, p <0.001), higher prevalence was predominantly observed among older children. The prevalence of *S*. *mansoni* was significantly higher among boys than girls (24.3% *versus* 18.7%; χ² = 29.9, p <0.001). The overall *S*. *mansoni* prevalence in treatment arms A, B, and C was 18.8% (95% CI: 17.2–20.3%), 20.5% (95% CI: 18.9–22.2%), and 27.2% (95% CI: 25.3–29.0%), respectively. With regard to the AM infection intensity, the respective values were 93.5 EPG (95% CI: 62.6–124.4 EPG), 96.2 EPG (95% CI: 74.5–117.9 EPG), and 88.1 EPG (95% CI: 71.5–104.7 EPG) (Table 1).

**Table 1:** *S*. *mansoni* and soil-transmitted helminth infection prevalence at the baseline and follow-up surveys, stratified by treatment arm.

Arms	Baseline				One-year post-treatment				Change
	Examined	Infected	Prevalence		Examined	Infected	Prevalence		
	N	N	(%)	(95% CI)	N	N	(%)	(95% CI)	(%)
**Arm A**									
*S*. *mansoni*	2,410	453	18.8	(17.2–20.3)	2,379	266	11.2	(9.9–12.4)	-40.4
Hookworm	2,410	16	0.7	(0.3–1.0)	2,379	2	0.1	(0.0–0.2)	-85.7
*A*. *lumbricoides*	2,410	23	0.9	(0.6–1.3)	2,379	11	0.5	(0.2–0.7)	-44.4
*T*. *trichiura*	2,410	82	3.4	(2.7–4.1)	2,379	42	1.8	(1.2–2.3)	-47.1
**Arm B**									
*S*. *mansoni*	2,348	482	20.5	(18.9–22.2)	2,288	332	14.5	(13.1–16.0)	-29.3
Hookworm	2,348	24	1.0	(0.6–1.4)	2,288	2	0.1	(0.0–0.2)	-90.0
*A*. *lumbricoides*	2,348	12	0.5	(0.2–0.8)	2,288	10	0.4	(0.2–0.7)	-20.0
*T*. *trichiura*	2,348	75	3.2	(2.5–3.9)	2,288	39	1.7	(1.2–2.2)	-46.9
**Arm C**									
*S*. *mansoni*	2,253	612	27.2	(25.3–29.0)	NA				
Hookworm	2,253	55	2.4	(1.8–3.1)	NA				
*A*. *lumbricoides*	2,253	31	1.4	(0.9–1.9)	NA				
*T*. *trichiura*	2,253	64	2.8	(2.2–3.5)	NA				
**Overall**									
*S*. *mansoni*	7,011	1,547	22.1	(19.5–24.4)	4,667	598	12.8	(11.9–13.8)	-42.1
Hookworm	7,011	95	1.4	(1.1–1.6)	4,667	4	0.1	(0.0–0.2)	-92.9
*A*. *lumbricoides*	7,011	66	0.9	(0.7–1.2)	4,667	21	0.4	(0.3–0.6)	-55.6
*T*. *trichiura*	7,011	221	3.2	(2.7–3.6)	4,667	81	1.7	(1.4–2.1)	-46.9

Prevalence of *S*. *mansoni* and soil-transmitted helminth infections among 9- to 12-year-old schoolchildren in the schools belonging to treatment arms A and B, respectively, at the baseline survey, which was conducted from December 2011 to February 2012, and the first follow-up survey, which was carried out one-year post-treatment in May 2013, in western Côte d’Ivoire.

Arm A: schools receive praziquantel treatment annually for four years, Arm B: schools receive praziquantel treatment in the first two years of the study, followed by two years of “drug holiday”; Arm C: schools receive praziquantel treatment in the first and third year of the study and have “drug holidays” in the second and fourth year.

CI: confidence interval; NA: not assessed.

### Changes of *S*. *mansoni* Prevalence at Follow-Up Survey

As summarized in Table 1, at the first follow-up survey, the overall *S*. *mansoni* prevalence in arms A and B showed a statistically significant decline from 19.7% (95% CI: 18.5–20.8%) at baseline to 12.8% (95% CI: 11.9–13.8%) at the 1-year follow-up. In arm A, a decrease from 18.8% (95% CI: 17.2–20.3%) to 11.2% (95% CI: 9.9–12.4%) was observed, corresponding to a reduction of 40.4%, while in arm B the prevalence declined from 20.5% (95% CI: 18.9–22.2%) to 14.5% (95% CI: 13.1–16.0%), a reduction of 29.3%.

**Fig 2:**
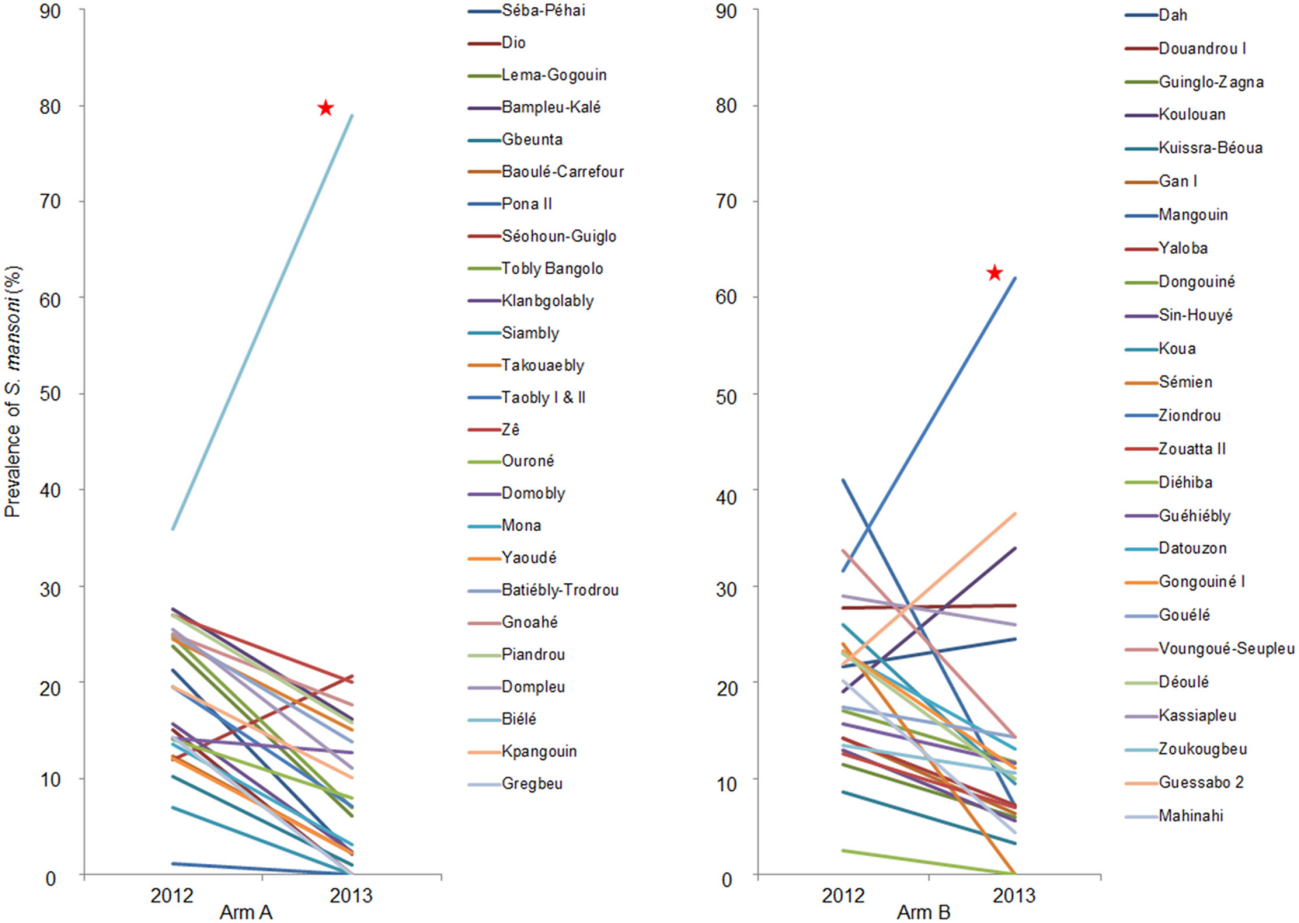
Dynamics of the *S*. *mansoni* prevalence in schools of treatment arms A and B. The graphs show the change of the *S*. *mansoni* prevalence from the baseline survey, which was conducted from December 2011 to February 2012, to the first follow-up survey, which was carried out one-year post-treatment in May 2013, in 9- to 12-year-old schoolchildren from 25 schools per treatment arm in western Côte d’Ivoire. Arm A: schools receive praziquantel treatment annually for four years, Arm B: schools receive praziquantel treatment the first two years of the study, followed by two years of “drug holiday”. Red star: *S*. *mansoni* prevalence increased significantly.

Fig 2 indicates the dynamics of the *S*. *mansoni* prevalence from baseline to first follow-up survey on a school-by-school basis, stratified by treatment arm. Among the 25 schools belonging to treatment arm A, the *S*. *mansoni* prevalence dropped in 23 schools (S1 Table). The most significant decreases occurred in Dio, Pona 2, Siambly, and Gregbeu, where at the 1-year follow-up, no eggs of *S*. *mansoni* were found in the stool of the children examined. However, in Biélé, the *S*. *mansoni* prevalence increased significantly from 36.0% (95% CI: 26.4–45.6%) to 79.0% (95% CI: 70.9–87.1%), while a non-significant increase from 12.0% (95% CI: 5.5–18.5%) to 20.7% (95% CI: 12.0–29.4%) was observed in Séohoun-Guiglo.

In treatment arm B, the prevalence of *S*. *mansoni* decreased in 20 out of the 25 schools included (S1 Table). In two schools, the prevalence dropped prominently to zero from 24.0% in Semien and from 25.6% in Diehiba. A significant increase in the *S*. *mansoni* prevalence was observed in Ziondrou from 31.6% (95% CI: 22.0–41.1%) to 62.0% (95% CI: 52.3–71.7%). An increase in prevalence was also observed in Dah, Douandrou 1, Koulouan, and Guessabo 2, but without statistical significance.

**Fig 3:**
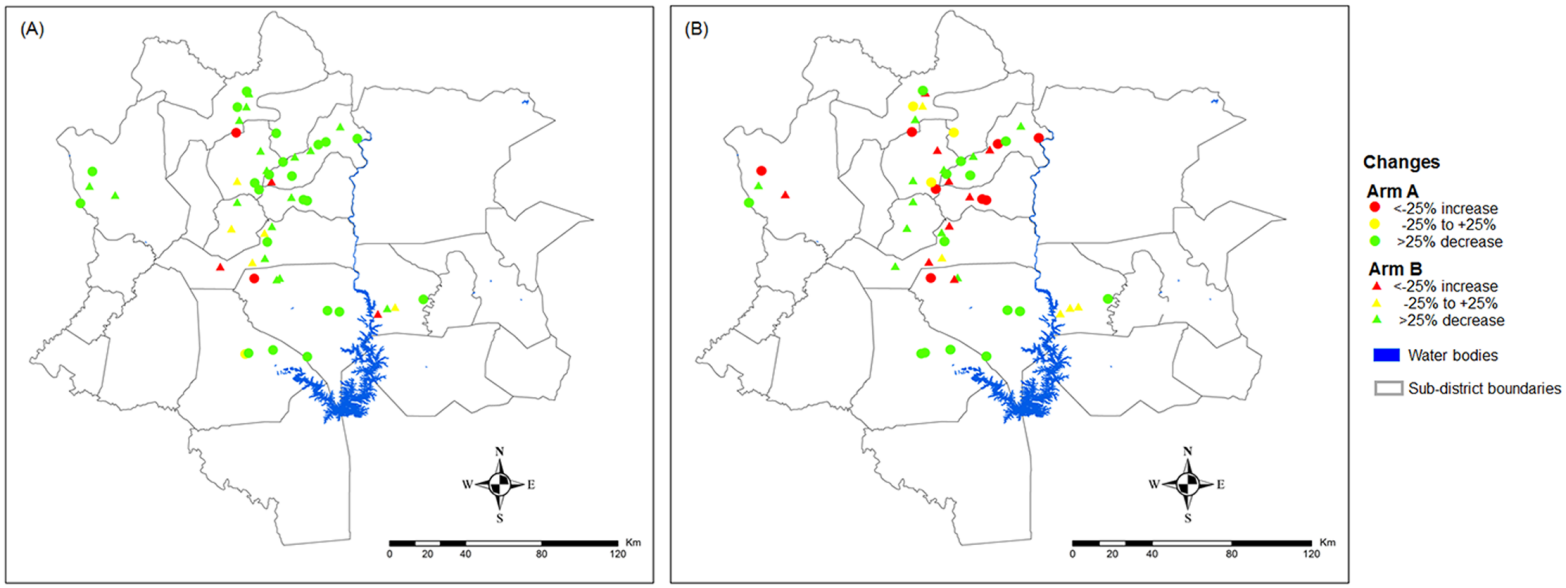
*S*. *mansoni* prevalence and infection intensity (AM EPG) at the baseline and follow-up survey. The maps show the spatial distribution of the changes in the *S*. *mansoni* prevalence and in the infection intensity expressed as arithmetic mean eggs per gram of feces (AM EPG) between the baseline survey (A), which was conducted from December 2011 to February 2012, and the first follow-up survey (B), which was carried out one-year post-treatment in May 2013, in western Côte d’Ivoire. Arm A: schools receive praziquantel treatment annually for four years, Arm B: schools receive praziquantel treatment the first two years of the study, followed by two years of “drug holiday”.

Taken together, as shown in Fig 3A, among the 50 schools surveyed at the first follow-up, a reduction of the *S*. *mansoni* prevalence of 25% and above was observed in 39 schools (78.0%). In six schools, the changes ranged from -25% to +25%. An increase of 25% and above was recorded in five schools (10.0%). The increase in prevalence was observed mainly in the central part of Guemon region, eastern Tonkpi region, and western part of Haut-Sassandra region.

### Changes of *S*. *mansoni* Infection Intensity at Follow-Up Survey

The overall *S*. *mansoni* AM EPG in arms A and B increased from 94.9 EPG (95% CI: 76.2–113.6 EPG) at baseline to 109.3 EPG (95% CI: 82.7–135.9 EPG) at the 1-year follow-up survey. However, this increase was not statistically significant. As shown in Table 2, in arm A, an increase from 93.5 EPG (95% CI: 62.6–124.4 EPG) to 123.7 EPG (95% CI: 70.7–176.7 EPG) was observed, corresponding to an increase of 32.3%, while in arm B the AM EPG at baseline (96.2 EPG, 95% CI: 74.5–117.9 EPG) and the 1-year follow-up (97.8 EPG, 95% CI: 75.5–120.0 EPG) remained basically the same. The proportion of children with heavy infections (≥400 EPG) increased from 4.9% to 6.3%.

**Table 2:** *S*. *mansoni* infection intensity in the schools belonging to treatment arms A, B, and C.

Arm	Baseline						One-year post-MDA						Change
	No.	No.	Arithmetic mean EPG	Light	Moderate	Heavy	No.	No.	Arithmetic mean EPG	Light	Moderate	Heavy	
	examined	positive	(95% CI)	(%)	(%)	(%)	examined	positive	(95% CI)	(%)	(%)	(%)	(%)
**Arm A**	2,410	453	93.5 (62.6–124.4)	85.9	9.5	4.6	2,379	266	123.7 (70.7–176.7)	79.3	13.5	7.2	-32.2
**Arm B**	2,348	482	96.2 (74.5–117.9)	81.1	12.9	6.0	2,288	332	97.8 (75.5–120.0)	75.6	18.7	5.7	-1.7
**Arm C**	2,253	612	88.1 (71.5–104.7)	77.1	18.8	4.1	NA	NA	NA	NA	NA	NA	
**Overall**	7,011	1,547	92.2 (79.2–105.3)	80.9	14.2	4.9	4,667	598	109.3 (82.7–135.9)	77.3	16.4	6.3	-18.5

*S*. *mansoni* arithmetic mean intensity of infection among 9- to 12-year-old schoolchildren in the schools belonging to treatment arms A, B, and C, respectively, as determined at the baseline survey, which was conducted from December 2011 to February 2012, and the first follow-up survey, which was carried out one-year post-treatment in May 2013, in western Côte d’Ivoire.

Arm A: schools receive praziquantel treatment annually for four years, Arm B: schools receive praziquantel treatment in the first two years of the study, followed by two years of drug holiday; Arm C: schools receive praziquantel treatment in the first and third year of the study and have “drug holidays” in the second and fourth year.

NA: not assessed; CI: confidence interval; EPG: eggs per gram of feces.

**Fig 4:**
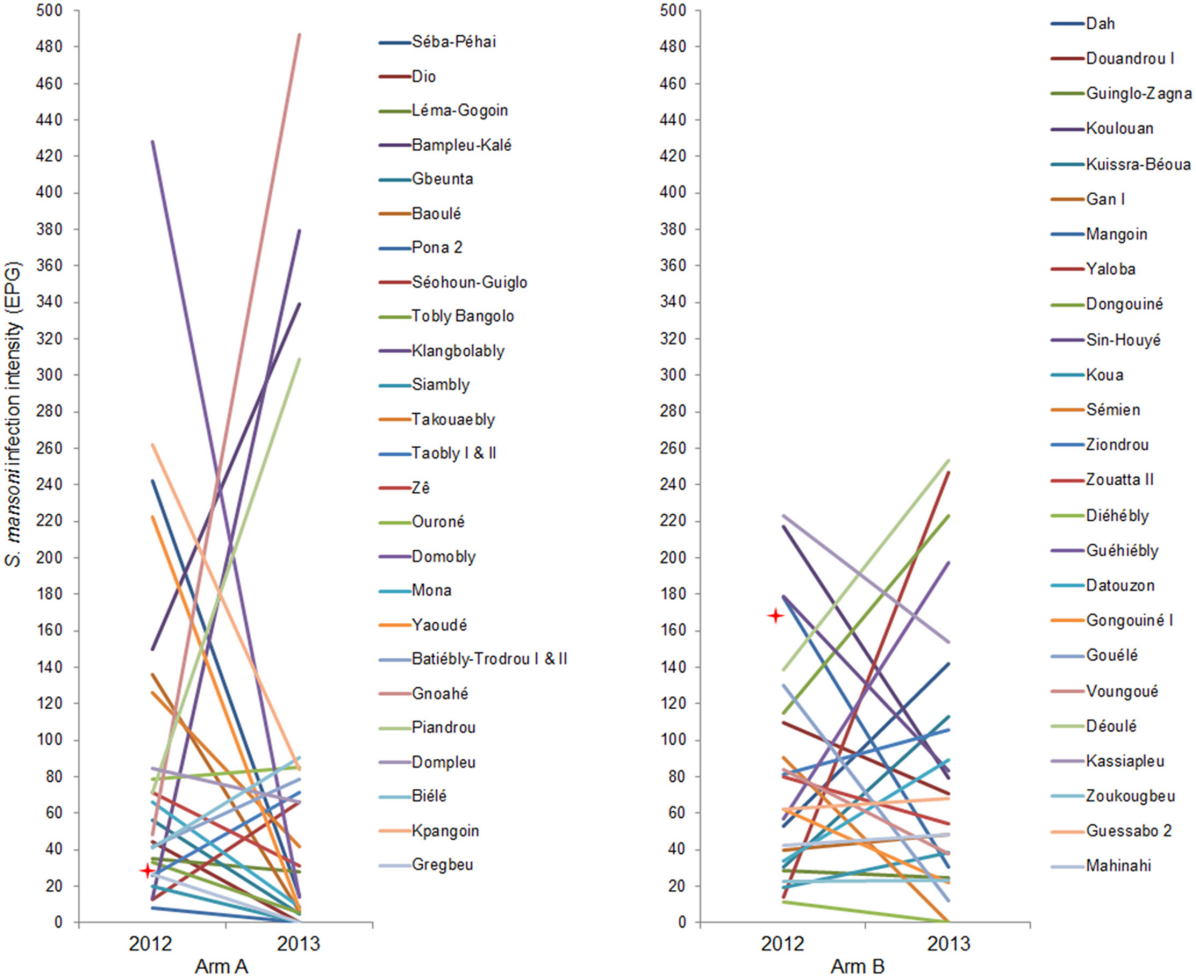
Dynamics of the *S*. *mansoni* infection intensity in schools of treatment arms A and B. The graphs show the change of the *S*. *mansoni* infection intensity expressed as change in arithmetic mean eggs per gram of feces (AM EPG) from the baseline survey, which was conducted from December 2011 to February 2012, to the first follow-up survey, which was carried out one-year post-treatment in May 2013, in 9- to 12-year-old schoolchildren from 25 schools per treatment arm in western Côte d’Ivoire. Arm A: schools receive praziquantel treatment annually for four years, Arm B: schools receive praziquantel treatment the first two years of the study, followed by two years of “drug holiday”. Red star: *S*. *mansoni* infection intensity decreased significantly.

Fig 4 displays the changes of the *S*. *mansoni* AM EPG in all the schools of treatment arms A and B from baseline to the first follow-up. In arm A, the *S*. *mansoni* AM EPG decreased in 16 (64.0%) out of the 25 surveyed schools (S1 Table). However, a statistically significant decrease in AM EPG from 33.0 EPG (95% CI: 13.9–52.0 EPG) to 5.5 EPG (95% CI: 3.8–7.2 EPG) was observed in only one school; Tobly Bangolo. Increases in *S*. *mansoni* AM EPG were observed in nine schools. However, the increase lacked statistical significance in all schools. In treatment arm A, the proportion of children with moderate (100–399 EPG) and heavy infections (≥400 EPG) increased from 9.5% to 13.5% and from 4.6% to 7.2%, respectively.

In arm B, a decrease of the *S*. *mansoni* infection intensity was observed in 13 (52.0%) out of the 25 schools (S1 Table). With the exception of one school, this decrease was not statistically significant. The AM EPG decreased significantly in Mangouin school from 178.0 EPG (95% CI: 77.7–278.3 EPG) to 30.4 EPG (95% CI: 4.2–56.6 EPG). In the remaining 12 schools, the AM EPG increased, but these increases lacked statistical significance. The proportion of children with moderate and heavy infection intensities increased from 12.9% to 18.7%, while the proportion of heavy infections decreased slightly from 6.0% to 5.7%.

Fig 3B shows the spatial distribution of *S*. *mansoni* AM EPG reduction after the intervention in the study area. The AM EPG decreased by at least 25% in 25 schools (50.0%). In eight schools (16.0%), the change varied from -25% to +25%. The AM EPG increased by 25% and more in 17 schools (34.0%). An increase of *S*. *mansoni* infection intensity by 25% and more was only focally observed; in Tonkpi region and central Guemon region.

### Coverage of SBT

During the SBT carried out in June 2012, the estimated number of the school-aged population in the study area was 31,832 children. Among them, 26,804 swallowed praziquantel tablets at the SBT, resulting in an overall DOT coverage of 84.2%. Stratified by treatment arm, we found a DOT coverage of 79.2% (range: 31.9–97.9%) for arm A, 84.8% (range: 61.5–98.5%) for arm B, and 88.4% (range: 75.1–98.9%) for arm C.

**Fig 5:**
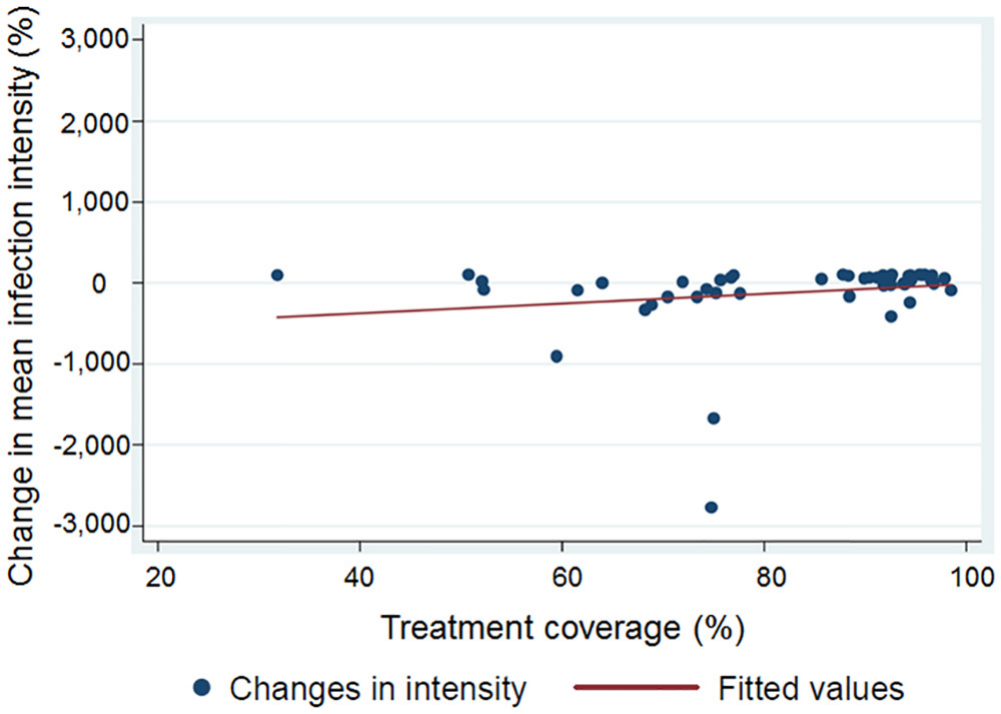
Correlation between coverage rate and the changes in the *S*. *mansoni* infection intensity. Scatter plot illustrating the correlation between the coverage rates achieved in a directly observed school-based treatment round implemented in 50 schools in western Côte d’Ivoire in June 2012, and the % changes in the *S*. *mansoni* arithmetic mean infection intensity observed between the baseline survey, which was conducted from December 2011 to February 2012, and the first follow-up survey, which was carried out one-year post-treatment in May 2013, in 9- to 12-year-old schoolchildren.

The individual DOT coverage rates achieved in the 75 villages are shown in S2 Table. A coverage of 75% and above was achieved in 57 schools (76.0%), while a coverage of less than 75% was reported in the remaining 18 schools. Yaoudé (in arm A) reported a coverage below 50%. The DOT coverage was not significantly correlated with changing levels of *S*. *mansoni* prevalence (Spearman ρ = -0.11; p = 0.43), while it was significantly correlated with AM EPG (Spearman ρ = 0.32; p = 0.02) (Fig 5).

## Discussion

Preventive chemotherapy with praziquantel is the backbone of the global strategy against schistosomiasis and other helminthiases [12,29]. Our findings show that one year after an initial treatment with praziquantel in 50 schools of western Côte d’Ivoire that met inclusion criteria of a SCORE harmonization protocol (prevalence ranging between 10% and 24%) [15], the overall *S*. *mansoni* prevalence was reduced from 19.7% to 12.8%, while there was no significant change in the overall AM EPG. The overall DOT coverage in the study area was 84.2%; hence, above the 75% coverage recommended by WHO [16]. At school level, the picture on the impact of the SBT was less clear cut. Decreases in prevalence and infection intensity were observed in some schools and increases in others. Among the six schools that showed higher prevalences of *S*. *mansoni* at the 1-year follow-up compared with baseline, in only one school, the treatment coverage was <75%. The changes in the AM EPG level were significantly correlated with the coverage rate.

The overall reduction of the *S*. *mansoni* prevalence in the first year of this SCORE project (35.0%) is in line with studies assessing the *S*. *mansoni* prevalence 12 months post-MDA in central Sudan and Uganda, where reductions of *S*. *mansoni* prevalence of 36.7% and 39.5% were observed, respectively [30,31]. The treatment coverage in these two studies was reported to be 100% and 79.2%, respectively [31,32]. In the Sudan study, treatment of children with praziquantel was conducted by trained nurses and medical officers, while in Uganda, the treatment was carried out by trained teachers and community drug distributors [31,32]. A survey conducted 6 months after praziquantel treatment in Sierra Leone where the overall treatment coverage was 94.0% found a reduction of the *S*. *mansoni* prevalence of 44.6% [33]. Another study carried out in Sierra Leone reported an even higher reduction in the *S*. *mansoni* prevalence of 67.2%, as determined three years after three rounds of praziquantel administration [34]. In contrast, studies conducted in Zambia and Kenya showed that 2 years after the withdrawal of praziquantel treatment led to an increase of *S*. *mansoni* prevalence [35,36]. It is important to note that these studies showed that the impact of MDA on the *S*. *mansoni* prevalence varied depending on the infection status in a given area, and the frequency and number of treatment rounds. Repeated treatments over short time periods can lead to a high reduction in *S*. *mansoni* prevalence compared to longer treatment intervals. Similar baseline *S*. *mansoni* prevalences were observed in two preceding studies in Sierra Leone and Uganda (49% and 42%, respectively), but the decrease in *S*. *mansoni* prevalence was lower in Uganda, where the intensity of infection, and thus the level of transmission, was higher. A plausible explanation of this observation arises from rapid re-infection, which is related to the force of infection, and which is likely higher where *S*. *mansoni* transmission is intense. Indeed, the level of schistosomiasis transmission, which is governed by various factors, such as local environmental determinants, climate, water contact patterns, intermediate host snail distribution, and ecology, may affect the impact of MDA [37–40]. When interpreting these results, one has to bear in mind, however, that the prevalence of *S*. *mansoni* was determined by an insensitive diagnostic approach; single stool samples subjected by single (Uganda) or duplicate Kato-Katz thick smears (Sierra Leone). Hence, the diagnostic approach was less rigorous than in the current study in Côte d’Ivoire, where only those children who had at least quadruplicate Kato-Katz thick smears examined before and after treatment were included in the final analysis.

In our study, in the schools Biélé and Ziondrou, the *S*. *mansoni* prevalence had significantly increased one year after SBT with levels in excess of 60%. Since the DOT coverage in both schools was high (75.2% in Biélé and 91.9% in Ziondrou), we assume that there are major transmission hotspots in the area, where children become rapidly re-infected. Re-emergence of *S*. *mansoni* and *S*. *haematobium* after treatment in high-endemicity areas has previously been reported from other studies in Côte d’Ivoire and Niger [41,42]. One explanation might be migration of people, including those infected with *S*. *mansoni* or *S*. *haematobium*, into treated villages. A considerable population movement has, for example, been observed in Côte d’Ivoire due to socio-political unrest in 2011 [43], hence at the start of our study. A lack of access to safe water, sanitation, and hygiene (WASH) might also be the reason for rapid reinfection. Noteworthy, when interviewing the local village leaders, they reported that people in the area frequently use well water for washing and bathing, while ponds and rivers serve as the main natural water contact sites. While some houses have latrines, many people still practice open defecation. Another explanation of the increase in *S*. *mansoni* prevalence might be the target population of the treatment strategy. The present study focused on school-aged children. Preschool-aged children and adults also harbor *Schistosoma* worms, and hence, they act as reservoir of transmission source of re-infections [36]. Yet, there are other local conditions that might foster *S*. *mansoni* transmission in Biélé and Ziondrou that warrant further investigation. For example, one might want to assess the frequency and duration of water contact in children and associated re-infection patterns, and the transmission force caused by intermediate host snails populating waterbodies located in close proximity to the surveyed schools. It will be important to assess in future surveys whether individuals had indeed received praziquantel in the past treatment round, or whether they were immigrating from other areas after the last survey, or had traveled to highly endemic areas over the past year. Ideally, the reinfection pattern would be determined by following a cohort of children, including immunological markers of the individuals that might favor or delay reinfection, and molecular markers of the infecting parasites.

An increase of *S*. *mansoni* infection within the frame of ongoing treatment programs has also been observed elsewhere. In Senegal, for example, an elevated *S*. *mansoni* prevalence was found 10 months after praziquantel administration [44]. More recently, in Ségou district in Mali, the national control program had revealed an increase of the *S*. *mansoni* prevalence after four rounds of MDA in 7- to 14-year-old children [45]. It has been assumed that these increases of *S*. *mansoni* infections after praziquantel treatment might be explained by partial resistance to praziquantel, the acquisition of new infection, and high force of transmission [46–48].

Taken together, our data show that SBT resulted in marked decreases of *S*. *mansoni* prevalence, but the intensity of infection among infected children did not change significantly. Hence, with a single treatment round, the force of transmission in terms of egg excretion in the school-aged population has not been changed in most of our study schools. Monitoring the impact of multiple treatment rounds and “drug holidays” over the next years will provide stronger evidence of what multiple SBT rounds can achieve [13,15].

Clearly, sustainable control and eventual elimination of schistosomiasis requires multiple intervention packages, such as preventive chemotherapy (perhaps extended to all age groups), intensified case management, control of intermediate host snails, provision of WASH, and setting-specific information, education, and communication (IEC) [49–51]. In Côte d’Ivoire, the control of schistosomiasis at a national scale is still at an early stage. Indeed, the PNL-SGF was only launched shortly before this SCORE project. For the success and sustainability of schistosomiasis control in Côte d’Ivoire–and elsewhere in sub-Saharan Africa–it will be important that, in addition to preventive chemotherapy, other control measures are considered and implemented [6,7,49,52].

The present study showed that one year after SBT with praziquantel, the overall prevalence of *S*. *mansoni* infection had decreased significantly. However, in certain hotspot schools, the *S*. *mansoni* prevalence had increased unexpectedly. The infection intensity among *S*. *mansoni*-infected children was similar at the 1-year follow-up. These results demonstrated that the dynamic of schistosomiasis in the study areas is heterogeneous and that a single round of treatment is insufficient to have a lasting effect. It will be important to monitor the dynamic of schistosomiasis over the course of this SCORE study, in order to deepen our understanding of the dynamics of schistosomiasis transmission in a moderately endemic setting.

## Supporting Information

S1 Table Treatment coverage, and changes in *S*. *mansoni* prevalence and intensity of infection in 50 schools in western Côte d’Ivoire from 2012 to 2013. (XLSX)

S2 Table Praziquantel coverage of the school-based treatment conducted in 75 schools in western Côte d’Ivoire in June 2012, stratified by intervention arm. (DOCX)

S1 Translation Translation of abstract into French. (DOCX)
