# Supplementary material for: Sustaining Control of Schistosomiasis Mansoni in Western Côte d’Ivoire: Results from a SCORE Study, One Year after Initial Praziquantel Administration
Source: PLoS Negl Trop Dis. 2016 Jan 20;10(1):e0004329. doi: 10.1371/journal.pntd.0004329 (PMC4720284; doi:10.1371/journal.pntd.0004329)
Supplement: S2 Table — (DOCX) [file pntd.0004329.s002.docx]

**Supporting Information**

**S2 Table.** Praziquantel coverage of the school-based treatment conducted in 75 schools in western Côte d’Ivoire in June 2012, stratified by intervention arm.

| **Treatment**  **arm** | **Village** | **Number of school-aged children** | **Number of school-aged children**  **treated by teachers** | **Coverage (%)** |
| --- | --- | --- | --- | --- |
| A | YAOUDE | 408 | 130 | 31.9 |
| A | GREGBEU | 835 | 424 | 50.8 |
| A | LEMA - GOGOUIN | 313 | 163 | 52.1 |
| A | BATIEBLY - TRODROU | 402 | 210 | 52.2 |
| A | GNOAHE | 242 | 144 | 59.5 |
| A | PIANDROU | 277 | 189 | 68.2 |
| A | TAOBLY | 549 | 403 | 73.4 |
| A | KLANBGOLABLY | 210 | 157 | 74.8 |
| A | BIELE | 206 | 155 | 75.2 |
| A | TAKOUAEBLY | 185 | 142 | 76.8 |
| A | BAOULE - CARREFOUR | 187 | 144 | 77.0 |
| A | BAMPLEU - KALE | 273 | 212 | 77.7 |
| A | TOBLY BANGOLO | 541 | 478 | 88.4 |
| A | GBEUNTA | 465 | 427 | 91.8 |
| A | KPANGOUIN | 484 | 448 | 92.6 |
| A | SEOHOUN GUIGLO | 338 | 313 | 92.6 |
| A | DIO | 218 | 202 | 92.7 |
| A | MONA | 602 | 568 | 94.4 |
| A | SIAMBLY | 766 | 731 | 95.4 |
| A | PONA II | 404 | 386 | 95.5 |
| A | DOMOBLY | 348 | 333 | 95.7 |
| A | SEBA - PEHAI | 180 | 174 | 96.7 |
| A | DOMPLEU | 367 | 355 | 96.7 |
| A | OURONE | 192 | 186 | 96.9 |
| A | ZÊ | 236 | 231 | 97.9 |
| B | DONGOUINE | 520 | 320 | 61.5 |
| B | ZOUKOUGBEU | 1731 | 1107 | 64.0 |
| B | KUISSRA - BEOUA | 337 | 232 | 68.8 |
| B | DAH | 505 | 356 | 70.5 |
| B | GUINGLO - ZAGNA | 221 | 159 | 71.9 |
| B | DEOULE | 347 | 258 | 74.4 |
| B | YALOBA | 216 | 162 | 75.0 |
| B | DOUANDROU I | 288 | 218 | 75.7 |
| B | SIN - HOUYE | 504 | 432 | 85.7 |
| B | SEMIEN | 929 | 816 | 87.8 |
| B | DATOUZON | 208 | 184 | 88.5 |
| B | VOUNGOUE- SEUPLEU | 567 | 510 | 89.9 |
| B | GONGOUINE 1 | 326 | 295 | 90.5 |
| B | KOULOUAN | 410 | 374 | 91.2 |
| B | MANGOUIN | 521 | 478 | 91.7 |
| B | ZIONDROU | 221 | 203 | 91.9 |
| B | GAN I | 713 | 660 | 92.6 |
| B | GUESSABO 2 | 1487 | 1395 | 93.8 |
| B | MAHINAHI | 298 | 280 | 94.0 |
| B | GUEHIEBLY | 754 | 712 | 94.4 |
| B | GOUELE | 218 | 206 | 94.5 |
| B | ZOUATTA II | 447 | 423 | 94.6 |
| B | DIEHIBA | 467 | 448 | 95.9 |
| B | KASSIAPLEU | 437 | 422 | 96.6 |
| B | KOUA | 671 | 661 | 98.5 |
| C | GUELEMOU | 299 | 166 | 55.5 |
| C | SEAMBLY | 357 | 245 | 68.6 |
| C | SIEBLY | 205 | 144 | 70.2 |
| C | KEITENAMBLY | 396 | 294 | 74.2 |
| C | GOHOUO - ZIBIAO | 405 | 304 | 75.1 |
| C | KIELE | 252 | 196 | 77.8 |
| C | GBABLASSO | 363 | 284 | 78.2 |
| C | GUIAPLEU | 143 | 113 | 79.0 |
| C | BAHE - BLAON | 253 | 206 | 81.4 |
| C | YEPLEU | 467 | 396 | 84.8 |
| C | TIENY - SIABLY | 198 | 174 | 87.9 |
| C | GBADROU | 251 | 221 | 88.0 |
| C | SINGOUIN | 241 | 223 | 92.5 |
| C | BLOLEU | 303 | 284 | 93.7 |
| C | GLAOU | 102 | 96 | 94.1 |
| C | ZAGOUE | 353 | 333 | 94.3 |
| C | GUINGLO - ZIA | 167 | 158 | 94.6 |
| C | TAHABLY - GLODE | 220 | 209 | 95.0 |
| C | DIBOBLY | 1651 | 1569 | 95.0 |
| C | FOUEDOUGOU | 667 | 644 | 96.6 |
| C | BEOUE | 683 | 667 | 97.7 |
| C | DOUMBIADOUGOU | 231 | 226 | 97.8 |
| C | PINHOU 1 | 298 | 292 | 98.0 |
| C | ZEO | 401 | 393 | 98.0 |
| C | GORODI | 355 | 351 | 98.9 |

Arm A: schools receive praziquantel treatment annually for 4 years, Arm B: schools receive praziquantel treatment in the first two years of the study, followed by two years of drug holiday; Arm C: schools receive praziquantel treatment in the first and third year of the study and have “drug holidays” in the second and fourth year.
