# Supplementary material for: Sustaining Control of Schistosomiasis Mansoni in Western Côte d’Ivoire: Results from a SCORE Study, One Year after Initial Praziquantel Administration
Source: PLoS Negl Trop Dis. 2016 Jan 20;10(1):e0004329. doi: 10.1371/journal.pntd.0004329 (PMC4720284; doi:10.1371/journal.pntd.0004329)
Supplement: S1 Translation — (DOCX) [file pntd.0004329.s003.docx]

**Lutte durable contre la schistosomiase à *Schistosoma mansoni* dans l'Ouest de la Côte d'Ivoire: résultats d'une étude du SCORE, un an après le traitement initial au praziquantel**

**Resumé**

**Introduction**

## Le consortium schistosomiase de recherche et d'évaluation opérationnelle (SCORE) a entrepris plusieurs études pour déterminer les meilleures stratégies pour le contrôle durable de la schistosomiase jusqu’à son l’élimination. En Côte d’Ivoire, une étude randomisée en grappes de 5 ans est en cours de réalisation dans 75 écoles et a pour objet de lutter durablement contre *Schistosoma mansoni*. Nous rapportons les niveaux d’infection de *S.* *mansoni* chez les enfants d’âge scolaire, un an après le traitement initial avec le praziquantel (TBS). Ces niveaux d’infection sont comparés avec les résultats de l’enquête de base pour déterminer l’impact de l’intervention initial.

**Méthodologie**

L’enquête de base (enquête transversale), a été conduite sur la période fin 2011 - début 2012. Durant cette enquête, 3 échantillons de selles ont été collectés chez les enfants âgés de 9 à 12 ans, dans 75 écoles. Le premier suivi a eu lieu en réalisé en mai 2013 et elle a conserné 50 écoles. Les échantillons de selles ont été soumis à deux lames de Kato-Katz. Le taux de couverture du traitement observé directement (TOD) a été évalué; la prévalence et l’intensité de l’infection à *S. mansoni* ont été comparées entre enquête de base et celle de suivi.

**Principaux résultats**

Dans les 75 écoles enquêtées, la prévalence de *S. mansoni* était de 22,1% (95% intervalle confidence (IC): 19,5-24,4%). Le taux de couverture du TOD a été de 84,2%. Dans les 50 écoles enquêtées pendant l’enquête de base et celle de suivi un an après traitement, la prévalence a baissé de 19,7% (IC 95 % : 18,5-20,8 %) à 12,8 % (IC 95 % : 11,9-13,8 %), tandis que la moyenne arithmétique du nombre d’œufs de *S. mansoni* par gramme de selle (OPG) de *S. mansoni* chez les enfants infectés a augmenté de 92,2 OPG (IC 95 % : 79,2-105,3 OPG) à 109,3 OPG (IC 95 % : 82,7-135,9 OPG). Dans deux des 50 écoles, la prévalence a augmenté considérablement, malgré le taux de couverture du TOD >75%.

**Conclusion/Importance**

Un an après le traitement initial à base scolaire, la prévalence de *S. mansoni* a baissé. Malgré cette tendance positive, une augmentation a été observée dans certaines écoles. De plus, l’intensité d’infection chez les enfants infectés a augmenté. Nos résultats soulignent l’hétérogénéité de la dynamique de transmission et constitue une donnée de référence pour les enquêtes de suivi annuel de cette étude de SCORE.
